# Targeted genetic therapies for inherited disorders that affect both cardiac and skeletal muscle

**DOI:** 10.1113/EP090436

**Published:** 2023-12-14

**Authors:** Yiangos Psaras, Christopher N. Toepfer

**Affiliations:** ^1^ Division of Cardiovascular Medicine Radcliffe Department of Medicine University of Oxford Oxford UK

**Keywords:** Duchenne muscular dystrophy, Friedreich's ataxia, gene therapy, Pompe disease, skeletal and cardiac muscle disease, therapeutics

## Abstract

Skeletal myopathies and ataxias with secondary cardiac involvement are complex, progressive and debilitating conditions. As life expectancy increases across these conditions, cardiac involvement often becomes more prominent. This highlights the need for targeted therapies that address these evolving cardiac pathologies. Musculopathies by and large lack cures that directly target the genetic basis of the diseases; however, as our understanding of the genetic causes of these conditions has evolved, it has become tractable to develop targeted therapies using biologics, to design precision approaches to target the primary genetic causes of these varied diseases. Using the examples of Duchenne muscular dystrophy, Friedreich ataxia and Pompe disease, we discuss how the genetic causes of such diseases derail diverse homeostatic, energetic and signalling pathways, which span multiple cellular systems in varied tissues across the body. We outline existing therapeutics and treatments in the context of emerging novel genetic approaches. We discuss the hurdles that the field must overcome to deliver targeted therapies across the many tissue types affected in primary myopathies.

## Abstract (highlights)

- **What is the topic of this review?**
  Overlapping disease pathomechanisms and therapeutic opportunities in neuromuscular, skeletal and cardiac muscle diseases in the context of novel genetic therapies.
- **What advances does it highlight?**
  This review outlines the diverse genetic changes that drive pathomechanism across a set of neuromuscular conditions and highlight the emerging targeted biological therapies that are being developed to treat these conditions, with additional discussion of the hurdles to actualising genetically targeted precision medicine.

## 1 INTRODUCTION

The muscles of the human body underlie many of the core functions of locomotion, breathing and heart function. Muscles are high‐energy‐usage tissues and require specialised and well‐controlled cellular homeostasis to ensure effective and durable function. The skeletal muscles of the body underlie human locomotion and respiration; when overused, they can regenerate. In contrast, the heart is a dual circulatory pump in constant use, and cardiomyocytes that comprise the heart cannot significantly regenerate or be endogenously replenished, a finding that took 20 years to establish. Therapeutic regeneration still remains a therapeutic goal in the field (He et al., 2020). There are many diseases that primarily affect tissues outside of the heart and skeletal muscle but also cause mixtures of primary and secondary myopathies because the processes that maintain homeostasis in these varying tissues overlap (Lynch & Farmer, 2021; Terman et al., 2008). Rare musculopathies often directly affect proteins that are expressed across both cardiac and skeletal muscle. These conditions tend to be progressively debilitating, impacting quality of life, and can become lethal due to cardiac or diaphragmatic involvement (Łoboda & Dulak, 2020). As treatments for the primary pathologies of these diseases progress and individuals live longer, the secondary cardiac pathologies become more pronounced (Łoboda & Dulak, 2020). Opportunities for developing novel targeted treatments have accelerated in the last decades as the genetic causes of these diseases have been unearthed. We discuss common paradigms of these rare multi‐system diseases with secondary cardiac involvement to highlight shared and diverse disease pathways and outline emerging gene‐targeted therapies.

## 2 GENETICS DEFINE ACTIONABLE TARGETS ACROSS INHERITED CONDITIONS THAT AFFECT MUSCLE

**FIGURE 1:**
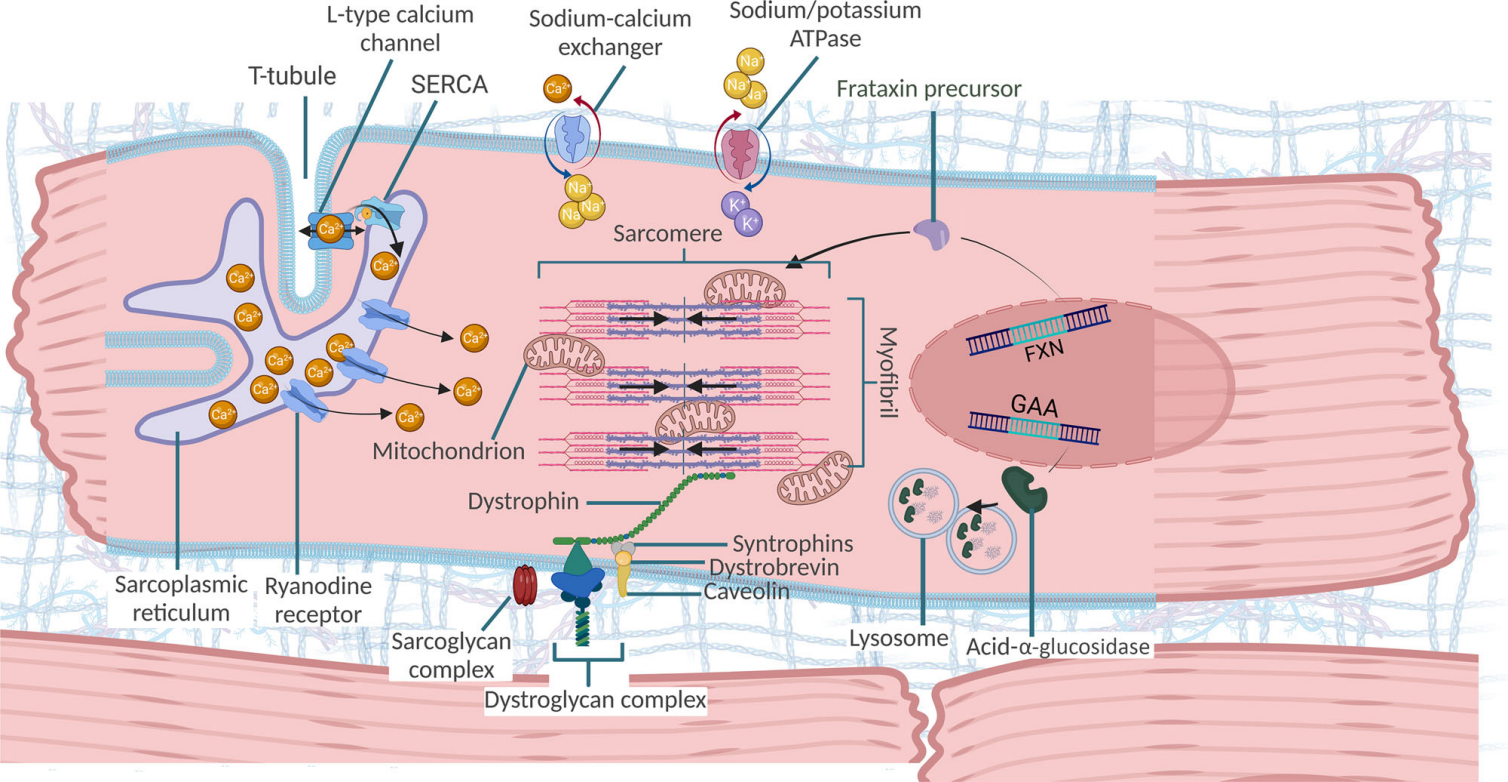
An overview of cardiomyocyte biology highlighting pathways altered in disease. The sodium/calcium exchanger and sodium/potassium ATPase mediate the entry of ions, depolarising the cell and triggering Ca^2+^ entry by the voltage‐gated L‐type calcium channels. This instigates calcium‐induced calcium release from the sarcoplasmic reticulum. Cytoplasmic calcium then binds the troponin complex allowing cross‐bridge formation and the ATPase driven myosin power stroke. The ATP used for these processes is supplied by the dense lattice of mitochondria packaged between sarcomeres. Mitochondrial function is supported by frataxin in complexes I–III, α‐glucosidase aids the supply of glucose for ATP production and dystrophin is integral to anchoring myofibrils to the sarcolemma allowing efficient force transduction between cells. Created using BioRender.com.

Myocytes are complex cells that work under tight homeostatic control. Dysregulation of fine processes that are integral to sustaining the core contractile function of myocytes precipitates pathology (Figure 1). Contraction is an energetically expensive process governed by transient Ca^2+^ fluxes that are propagated through excitation–contraction coupling and calcium‐induced calcium release. These processes propagate the synchronised release of calcium from intracellular calcium stores allowing cross‐bridge formation and force production by the myosin ATPase‐dependent power stroke, which is then transduced along myofibrils to neighbouring cells. This process is repeated approximately 70 times per minute in the adult heart consuming 6 kg of ATP daily (Neubauer, 2007).

Many of the homeostatic processes of the myocyte that are integral to maintaining contraction are shared across a variety of cell types. This overlap is especially evident in musculopathies, as many highly expressed genes overlap in cardiac, skeletal and smooth muscle. Therefore, rare variants of these overlapping genes can often affect multiple tissues. In this context we discuss a sub‐set of diseases that have cardiac manifestations which develop alongside other tissue pathologies. Many of these conditions have well‐established or emerging genetic mechanisms which provide opportunities for designing targeted genetic therapies for the multi‐tissue diseases they precipitate. There are several hurdles that will need to be overcome to produce a working gene therapy that can be effectively delivered to affected tissues. The last decade has seen the development of a variety of novel gene manipulation tools that can be used to design therapies. These include direct gene delivery systems packaging mRNA, genome editing techniques such as base‐editing (Dunbar et al., 2018; Rees & Liu, 2018) and prime editing (Scholefield & Harrison, 2021), CRISPR regulation in the form of CRISPR activation (CRISPRa) or inhibition (CRISPRi) (Kan & Doudna, 2022), anti‐sense oligonucleotides for altering translation, short interfering RNA (siRNA) and microRNA (miRNA) approaches for specific gene knockdown (Dana et al., 2017). We discuss these varied therapeutic strategies in relation to three specific diseases that have diverse genetic causes that all lead to a loss of gene product. These are Duchenne muscular dystrophy (DMD), a muscular dystrophy altering dystrophin expression, Friedrich's ataxia (FA), a rare neuromuscular condition reducing frataxin expression, and Pompe disease, a lysosomal storage disorder driven by acid α‐glucosidase deficiency. We discuss the existing non‐genetic therapies used as treatments for these diseases and outline the opportunities and hurdles to developing novel targeted genetic therapies for these conditions.

## 3 DUCHENNE MUSCULAR DYSTROPHY

DMD is a severe progressive X‐linked autosomal recessive disorder affecting 1 in 3500–10,000 people (Mah et al., 2014). DMD is characterised by progressive muscle weakness in early childhood. Cardiomyopathy typically presents in the mid‐teens with progressive fibrosis, left ventricular dysfunction, dilatation and heart failure (Kaspar et al., 2009). There is a significant mortality associated with DMD cardiomyopathy, where 41.9% of carriers die of cardiac complications at a mean age of 26 years, and there is a clear lack of targeted therapies to halt or reverse cardiac complications in these patients (Wahlgren et al., 2022).

Genetically DMD is driven by mutations in the *Dp71* and *Dp427* genes that encode cardiac and skeletal dystrophin. Sixty‐four percent of DMD cases are caused by deletions, 11% by duplications and 23% by substitutions (point mutations, insertions and small deletions), where as many as 30% of these variants are *de novo*, as reported in the Leiden Open Variation Database (https://databases.lovd.nl/shared/genes/DMD). Mutations cluster at exons 2–10 and 45–55, terminating the protein at the F‐actin binding domain or cysteine‐rich domain, respectively, resulting in truncated proteins which disrupt the link between cytoskeleton and extracellular matrix (Ankala et al., 2012; White et al., 2006).

**FIGURE 2:**
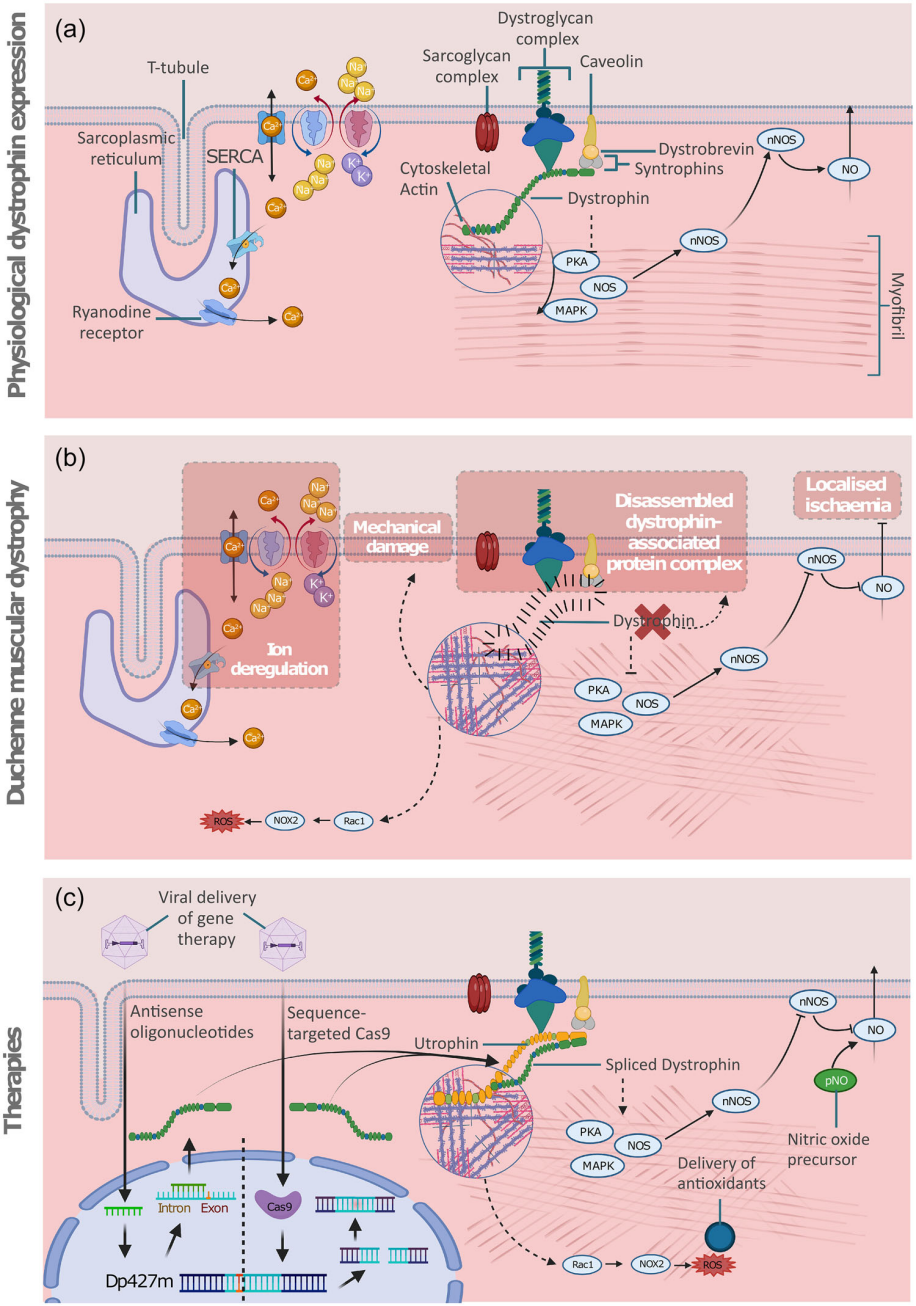
Dystrophin, Duchenne muscle dystrophy and therapeutic opportunities. (a) Physiological expression of dystrophin maintains cellular homeostasis and ion exchange by mechanical association of the dystrophin‐associated protein complex (DAPC), which includes the dystroglycan and sarcoglycan complexes, to the mechanical apparatus via cytoskeletal actin. This in turn links to the mitogen‐activated protein kinase (MAPK) and protein kinase A (PKA) pathways and nitric oxide synthase (NOS) signalling, enabling downstream signalling, correct localisation of neuronal NOS (nNOS) and subsequent secretion of vasodilative nitric oxide (NO). (b) Loss of dystrophin disrupts myofibril mechanical linkage to the sarcolemma, causing the DAPC to disassemble causing membrane damage. Loss of dystrophin‐mediated anchoring of cytoskeletal components interrupts PKA and MAPK signalling and causes myofibril disarray. Contractile processes activate Rac1 signalling, resulting in reactive oxygen species (ROS) damage. Loss of NOS signalling results in localised ischaemia due to mislocalisation of nNOS and a subsequent lack of NO secretion in the local vasculature. (c) Current therapeutic approaches include antisense oligonucleotides and gene editing strategies to generate spliced dystrophin. Others include supplementing expression of the dystrophin analogue utrophin, antioxidants to ameliorate ROS damage, or delivery of NO precursors to partially recover dystrophin function to ameliorate cell damage. Created using BioRender.com.

Dystrophin localises at the sarcolemma and mediates attachment to the extracellular matrix via interactions with cytoskeletal elements, ion channels and signalling proteins forming the dystrophin‐associated protein complex (DAPC) (Figure 2). Cardiac dystrophin binds to α‐actinin and interacts with cardioprotective AHNAK1, Cavin‐1, α‐crystallin B chain (CRYAB) and Cypher (Johnson et al., 2012). In DMD the partial or complete loss of dystrophin results in disassembly of the DAPC, which disrupts anchoring of the contractile apparatus to the cell membrane. This interrupts mechanical stress handling at the sarcolemma, rendering it susceptible to damage during contraction (Dudley et al., 2006). There are several other affected cellular processes summarised in Figure 2b.

Existing non‐targeted therapies have shown promise in treating DMD‐associated cardiomyopathy. These block pro‐inflammatory genes non‐specifically by inhibiting nuclear factor‐κB (NF‐kB) (Finkel et al., 2021) or hematopoietic‐type prostaglandin D synthase (HPGDS) (Mohri et al., 2009) (NCT02246478, NCT02752048). Suppression of the pathological mediator of hypertrophy and fibrosis TRPC6 (transient receptor potential cation channel, subfamily C, member 6), using BI749327 has shown efficacy in reducing cardiac fibrosis and increasing muscle function, abating the pro‐fibrotic response in the DMD mice and significantly increasing survival (Lin et al., 2022).

Genetically ameliorating the dystrophin deficiency in DMD is an attractive therapeutic goal to prevent downstream sequelae of the disease, negating the need for multiple tandem therapies. Increasing dystrophin expression is becoming methodologically tractable due to advances in biologics as deliverable therapies (Kupatt et al., 2021). The varied genetic aetiology of DMD complicates the use of patient‐specific genetic corrections, and unfortunately full‐length dystrophin is too large to be packaged into current vectors for direct delivery (Pichavant et al., 2011), but shorter, spliced functional dystrophin has been shown to prevent fibrosis and increase muscle function in *mdx* mice (Potter et al., 2021). Gene editing with adeno‐associated virus (AAV) delivery of CRISPR–Cas9 has been efficacious in mice, where removal of the early termination codon of dystrophin in the DMD model has shown partial correction of dystrophin expression, rescuing phenotype in cardiac and skeletal muscle (Long et al., 2016). Exon skipping can be used to partially circumvent out‐of‐frame mutations that reduce dystrophin expression, alleviating the phenotype (Aartsma‐Rus et al., 2009). Similarly, antisense oligonucleotides (ASOs) can be used to skip exons with premature stops and increase dystrophin levels (NCT00844597, NTR1241) (Table 1). Alternatively, boosting utrophin, a highly homologous dystrophin analogue, resulted in higher resistance to physical stress and reduced muscle damage (Tinsley et al., 2011); however conflicting data have shown a lack of efficacy in this approach (Muntoni et al., 2017). A small molecule, ataluren, which aids read‐through of premature termination codons, has been shown to partially restore dystrophin expression (Welch et al., 2007). Unfortunately, ataluren only functions on early termination of translation mutations caused by missense variants, which restricts its utility to a small subset of patients. This same principle applies to gene editing techniques that target specific exons, as the diversity of gene mutations that precipitate DMD is large. Therefore, each targeted editing approach would only provide a therapy for a subset of DMD patients.

**TABLE 1:** Completed and ongoing clinical trials for Duchenne muscular dystrophy.

Trial number	Intervention	Findings	Limitations	Ref.
NCT03703882	NF‐κB inhibition (edasalonexent)	Decreased functional decline in basic mobility and mild improvement in upper limb functional outcomes in patients under 6 years of age	No improvements in older patients	Finkel et al. (2021)
NCT02246478	Inhibitor of HPGDS (TAS‐205)	Reduced inflammatory mediator prostaglandin D2 (indicating increased muscle regeneration)	Small sample size, no functional assessment	Takeshita et al. (2018)
NCT02752048	Inhibitor of HPGDS (TAS‐205)	Reduced loss of muscle volume	No changes in mobility achieved	Komaki et al. (2020)
NCT01995032	Nitric oxide precursors (metformin and l‐citruline)	Slowed muscle degeneration	Small sample size and higher‐than‐expected variability may have affected statistics	Hafner et al. (2019)
NCT03354039	Antioxidant (tamoxifen)	Currently ongoing, no data published	—	Nagy et al. (2019)
NCT03375164	AAV delivery of micro‐dystrophin	Increased expression of micro‐dystrophin and DAPC components indicating partial recovery of the dystroglycan complex, improved ambulatory outcomes, reduced creatine kinase	Extremely limited sample size, no placebo controls used, no functional assessment	Mendell et al. (2020)
NCT00844597	Exon skipping	Increased dystrophin expression, increased expression of DAPC proteins α‐sarcoglycan and nNOS, reduced inflammatory infiltrates	Only 7 out of 19 patients responded to treatment, no functional assessment	Cirak et al. (2011)
NTR1241	Exon skipping	Increased dystrophin expression, increased patient mobility	—	Goemans et al. (2011)
NCT02858362	Utrophin modulation (ezutromid)	Terminated, data not reported	No efficacy reported in utrophin expression, functional outcomes and muscle regeneration	Muntoni et al. (2017)

## 4 FRIEDRICH'S ATAXIA

FA is the most common hereditary ataxia, affecting 1 in 25,000–50,000 individuals. It typically presents between 5 and 15 years of age where progressive ataxia leads to a reduction and eventual loss of mobility. Although FA is primarily a neuromuscular disease typified by muscle weakness (Fichera et al., 2022), it also precipitates a severe cardiomyopathy (Fichera et al., 2022; Weidemann et al., 2012). FA is an autosomal recessive condition caused by an expansion of a trinucleotide GAA repeat element in intron 1 of the frataxin gene (*FXN*) (Campuzano et al., 1996). Expansion ranges from 66 to 1700 repeats and reduces *FXN* gene expression, where longer repeat elements correlate with lower *FXN* expression and increased disease severity (Dürr et al., 1996; Schöls et al., 1997). Ninety‐six percent of FA cases have 4–29% of regular protein levels (Campuzano et al., 1997).

**FIGURE 3:**
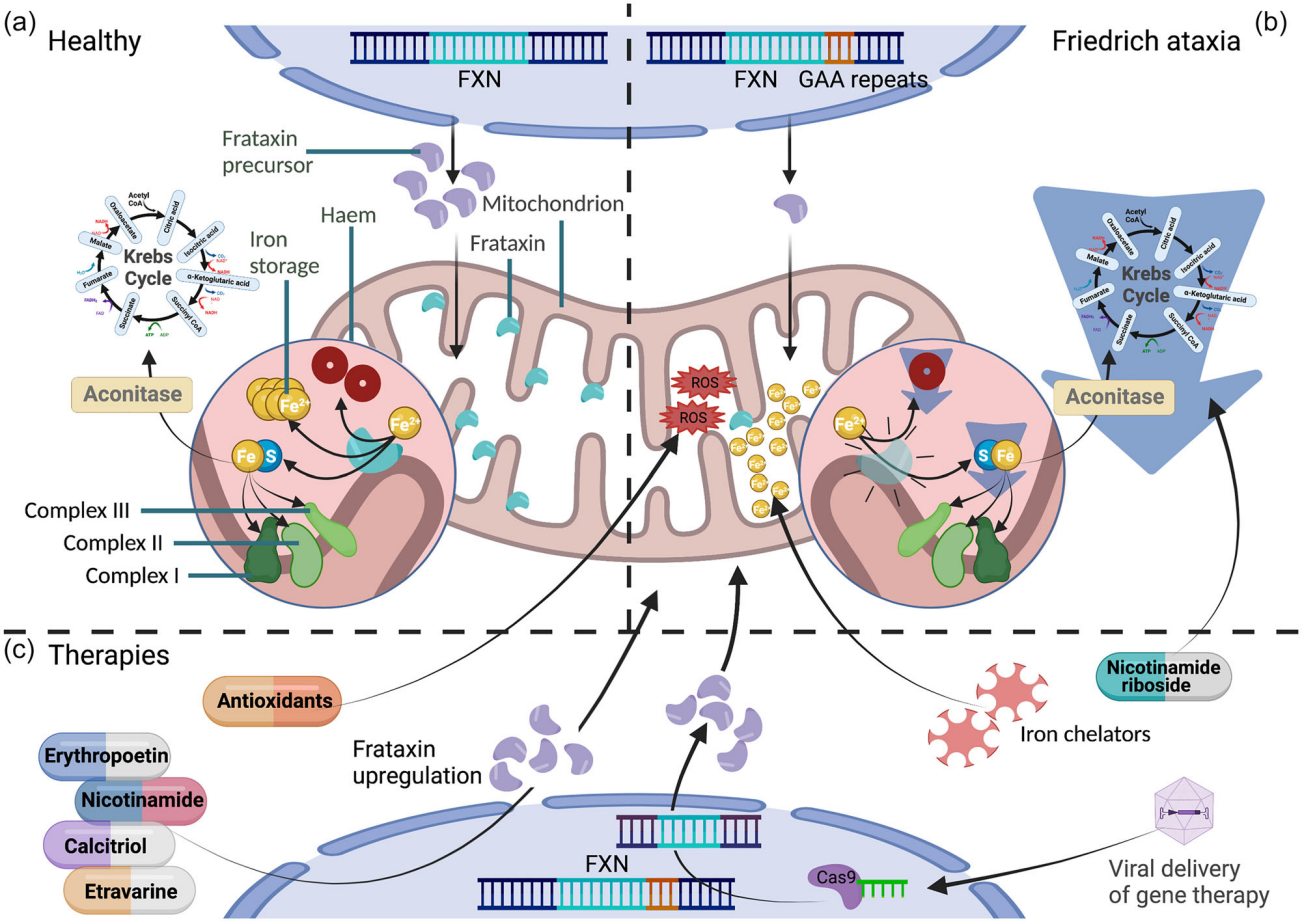
The role of frataxin in healthy and FA cardiomyocyte, highlighting opportunities for therapeutic intervention. (a) Expression of frataxin supports mitochondrial function by mediating iron–sulphur cluster formation, which is a key component of respiratory chain complexes I, II and III, a cofactor to aconitase, essential in the Krebs cycle, and contributes to haem formation and iron storage. (b) Decreased frataxin expression interferes with ATP production by reducing aconitase activity causing the interruption of the respiratory chain complexes I–III. Deregulation in iron metabolism results in iron accumulation, decreased haem production and ROS damage. (c) Therapeutic strategies include antioxidants against ROS damage, re‐purposing of FDA approved drugs that increase frataxin expression, iron chelation to counter iron accumulation, and supplementation with nicotinamide riboside to boost NAD^+^ and protect against the metabolic vacuum in FA cardiomyopathy. Gene therapy approaches aim to target and cleave the expanded GAA repeat with the aim of increasing *FXN* expression. Created using Biorender.com.

Frataxin is abundant in mitochondria (Gakh et al., 2010) and expressed highly in the central nervous system (CNS), pancreas, heart, liver and skeletal muscle, mirroring the tissue penetrance of FA's pathology. It is thought to be essential in the assembly of Fe–S clusters in mitochondria (Fox et al., 2019), which are involved in mitochondrial respiration via complexes I, II and III, and are cofactors for aconitase in the Krebs cycle; thus a loss of frataxin reduces ATP production (Figure 3). Frataxin is also implicated in iron metabolism, transport and storage, and mitochondrial biogenesis (Llorens et al., 2019). Decreased cellular frataxin causes iron accumulation in the mitochondria and depletion in the cytosol in yeast models (Babcock et al., 1997), which is reversible with re‐expression of frataxin (Radisky et al., 1999). Frataxin is also involved in apoptosis via iron deregulation (Wong, 1999), and oxidative stress defence (Shoichet et al., 2002).

FA cardiomyopathy has a heterogeneous presentation and can present as interventricular septum, left ventricle (LV) or concentric hypertrophy (Meyer et al., 2007). The LV wall is known to lose mass, exhibiting progressive fibrosis, arrhythmias and eventual heart failure (Weidemann et al., 2015). The mechanism of disease in FA cardiomyopathy is not fully understood, but it shows many of the hallmarks of inherited cardiomyopathies including irregular mitochondria, prolonged action potentials, and systolic and diastolic insufficiency (Hick et al., 2013; Wong et al., 2019). What sets FA cardiomyopathy apart from other cardiomyopathies is iron accumulation in the myocardium, myocarditis (Koeppen et al., 2015) and energy depletion driven by reduced ATP production (Lodi et al., 2001). This is different from hypertrophic cardiomyopathy (HCM), where energy depletion is driven by overactive myosin depleting ATP (Ashrafian et al., 2003; Margara et al., 2022; Schmid & Toepfer, 2021; Toepfer et al., 2020). Notably mitochondrial deficit conditions phenotypically imitate HCM suggesting that energy depletion is a central player in FA cardiomyopathy (Ashrafian et al., 2003).

Therapy in FA is multifaceted, reflecting its multisystem involvement, and ranges from exercise and physiotherapy (Widener et al., 2020) to bracing or surgical correction, muscle relaxants for muscle spasms, insulin therapy for diabetes, and interventions against sight and hearing loss (Corben et al., 2014). Cardiac symptoms are generally treated with angiotensin converting enzyme inhibitors and beta‐blockers, where severe cases sometimes require implantable cardioverters. Antiarrhythmics are used in instances of atrial fibrillation and transplant can be necessary in end‐stage heart failure (Corben et al., 2014). Therapies being investigated (Table 2) target sequelae of FA pathology by salvaging mitochondrial function, upregulating frataxin expression, increasing iron chelation (Kakhlon et al., 2008; Velasco‐Sánchez et al., 2011), or antioxidant treatment to limit damage (Qureshi et al., 2021). To date, one drug has gained US Food and Drug Administration (FDA) approval (omaveloxolone) and this showed improvements in the modified Friedrich's Ataxia Rating Scale (Lynch et al., 2021). Benefits in neurological function have been shown with nicotinamide (Libri et al., 2014); its combination with exercise therapy is being explored to compensate the metabolic deficit in FA. The peroxisome proliferator‐activated receptor γ agonist leriglitazone is also being investigated, as it restores the mitochondrial membrane potential, improving the mitochondrial deficit observed in models of FA (Rodríguez‐Pascau et al., 2021). Calcitriol has been shown to increase frataxin expression and restore mitochondrial membrane potential in neurons, cardiomyocytes and lymphoblastoid cells from FA patients (Britti et al., 2021). Etravirine, an FDA‐approved antiviral indicated for HIV treatment, has been shown to enhance frataxin expression in patient‐derived cells prompting a phase II clinical trial in patients (Alfedi et al., 2019). Erythropoietin, another FDA‐approved drug, has also been shown to increase frataxin expression in neurones, lymphocytes and cardiomyocytes (Sturm et al., 2005).

**TABLE 2:** Completed and ongoing clinical trials for Friedreich ataxia.

Trial number	Intervention	Findings	Limitations	Ref.
NCT04192136	NAD^+^ precursors combined with exercise	Ongoing, no results reported	—	—
NCT01589809	HDAC (histone deacetylase) inhibition to combat FXN silencing	Upregulation of frataxin, no changes in clinical assessment of functionality	No long‐term follow‐up (8 weeks)	Libri et al. (2014)
NCT02660112	Antioxidants (epicatexin)	No improvement in neuromuscular function (dexterity and mobility tests), improved cardiac outcomes (increased LV ejection fraction, increased septal thickness, reduced LV mass)	Small cohort, no control group used	Qureshi et al. (2021)
NCT00537680	Antioxidants (idebenone)	No changes in neuromuscular function	Short term study (6 months)	Lynch et al. (2010)
—	Antioxidants (idebenone)	Improved cardiac outcomes (reduced LV mass), mixed findings in the cohort on shortening fraction	Indications of improved dexterity not investigated, short term study (6 months)	Hausse et al. (2002)
NCT04801303	Calcitriol	Currently ongoing, no results reported	—	—
—	Combined antioxidant and iron chelation treatment (idebenone, deferiprone)	Improved functional parameters (posture, gait, mobility), reduced hypertrophy (septal thickness, LV mass), reduced iron in the cerebellar dentate nucleus		Velasco‐Sánchez et al. (2011)
—	Etravarine	Currently ongoing, no results reported	—	Alfedi et al. (2019)
Pre‐clinical	Sensory enhancement (balance‐based torso weighting)	Increased functionality (duration of standing), moderate correlation between severity of ataxia and improvement	No movement speed improvement observed	Widener et al. (2020)
NCT00631202	Erythropoietin	Increased frataxin expression (delayed), transient (<1 month) reduction in serum iron, no clinical improvement	Very small cohort	Saccà et al. (2011)
NCT05445323 and NCT05302271	Gene therapy – delivery of *FXN* with adeno‐associated virus (AAVrh.10hFXN)	Ongoing, no results reported	—	—

Therapeutic strategies to increase frataxin expression directly target the loss of cellular frataxin that drives FA pathology. Reduced cellular frataxin is known to alter the transcriptome, which is not completely overcome by re‐expression (Li et al., 2015), suggesting a developmental deficit in FA that may persist with treatment in adulthood. Re‐expression of frataxin is further complicated by the observation that supranormal levels of frataxin can drive cardiac pathology (Belbellaa et al., 2020), complicating the use of direct gene delivery due to potential cell specific overexpression leading to toxicity. Genetic correction of the *FXN* allele is therefore particularly attractive as *FXN* expression would remain under the control of endogenous gene regulatory elements. Encouraging preclinical work has shown that transplantation of CRISPR/Cas‐9 corrected FA progenitor and haematopoietic stem cells corrects host frataxin expression (Rocca et al., 2020). Such work highlights the potential for both autologous transplantation of gene‐corrected cells and in vivo excision of the expanded GAA repeat elements using CRISPR/Cas‐9 in endogenous cells. One complication in such a therapy is delivery to both the CNS and the heart. Two very recent clinical trials (NCT05445323 and NCT05302271) follow this design rationale to specifically address cardiomyopathy in FA by delivering the *FXN* gene via an AAVrh10 vector with specific tropism to muscles.

## 5 POMPE DISEASE

Pompe disease is an autosomal recessive condition affecting approximately 1 in 40,000. It is a lysosomal storage disease, prominently affecting skeletal muscle and the heart. It usually presents as one of two distinct manifestations: the early onset ‘classical’ presentation which occurs within a few weeks of life, displaying developmental abnormalities, progressive muscle weakness, low muscle definition and prominent cardiac involvement (van den Hout et al., 2003), and the late‐onset disease typified by muscular symptoms and a less prevalent cardiomyopathy (Boentert et al., 2016).

**FIGURE 4:**
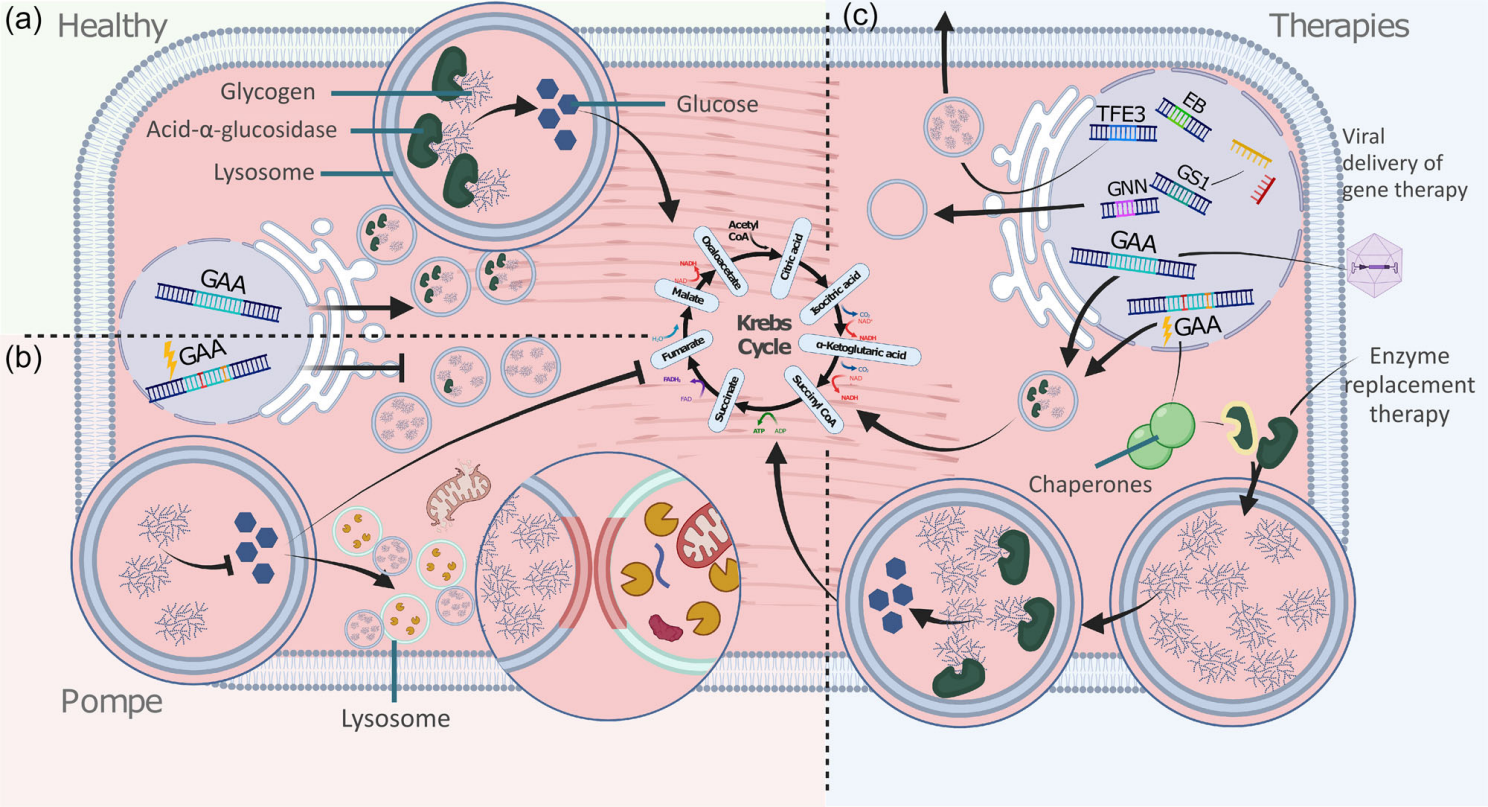
The role of acid α‐glucosidase in Pompe disease, and therapeutic strategies. (a) At physiological expression levels acid α‐glucosidase localises in lysosomes and hydrolyses glycogen to glucose for respiration. (b) A deficiency of acid α‐glucosidase in the cell caused by mutations in *GAA* results in accumulation of lysosomal glycogen and an inability to fuse lysosomes to phagosomes, causing a build‐up of autophagic material, a lack of organelle clearance, metabolic deficiency and loss of sarcomeres. (c) Therapeutic approaches include enzyme replacement therapy to complement the lack of functional acid α‐glucosidase, delivery of chaperones to stabilise the native and delivered acid α‐glucosidase, and viral delivery of wild‐type *GAA* gene or precursor acid α‐glucosidase. Alternatives include the overexpression of TFE3 and EB to induce secretion of lysosomal contents and RNA interference of glycogen synthase 1 (GS1) and glycogenin (GNN) to limit the production of glycogen. Created using BioRender.com.

Pompe disease is caused by mutations in the *GAA* gene which cause either a complete or partial deficiency of its gene product, acid α‐glucosidase, a lysosomal enzyme catalysing the hydrolysis of glycogen, the main glucose storage molecule. To date, over 580 mutations have been identified in *GAA*; complete deficiency causes early onset disease, while partial deficiency correlates with the less severe late onset disease (Peruzzo et al., 2019). *GAA* mutations can result in incorrect splicing, exon skipping or early termination reducing gene product. This is associated with the accumulation of glycogen in lysosomes, which become enlarged (Canibano‐Fraile et al., 2023). Phagosomes are unable to fuse with the lysosomes, causing a build‐up of autophagic material (Figure 4) (Raben et al., 2012). Cellular damage follows due to the cytosolic release of lysosomal contents, metabolic insult and inhibition of autophagy. Autophagy plays a major role in decreasing oxidative stress by removing compromised mitochondria to maintain efficient ATP production (Ju et al., 2016). Pompe's cardiac phenotype is thought to be driven by alterations in fatty acid metabolism (Yang et al., 2019), alongside general cellular energy deficits (Ashrafian et al., 2003), leading to a HCM phenocopy.

The only currently approved treatment for Pompe disease is enzyme replacement therapy (ERT), where recombinant acid α‐glucosidase is delivered to hydrolyse glycogen and reverse its accumulation (Thurberg et al., 2006). Longitudinal studies have shown that ERT has variable long‐term clinical efficacy based on disease presentation. There is little cardiac involvement in patients with late‐onset disease and ERT has been found to stabilise respiratory function and muscle strength, and attenuate the muscle phenotype in these patients (Sarah et al., 2022). In infantile onset Pompe, which has a prominent cardiomyopathy, long‐term improvements have been seen including the amelioration of hypertrophic cardiac remodelling and normalisation of cardiac function (Scheffers et al., 2023). Improvements in cardiac manifestations of the disease have meant that patients survive longer and begin to exhibit cognitive function changes, suggesting incomplete correction by ERT, due to a lack of delivery through the blood–brain barrier (van Gelder et al., 2012). Immune clearance and poor muscle delivery limit ERT efficacy (Bronsema et al., 2015). This has been approached by developing a novel enhanced recombinant acid α‐glucosidase with stabilising chaperones, which has shown efficacy in mice but did not improve outcomes over existing ERT therapy in phase III clinical trials (Schoser et al., 2021; Xu et al., 2019).

Approaching the treatment of Pompe disease with targeted gene therapies is attractive as restoration of a fully functional *GAA* gene would eliminate the haploinsufficiency that drives Pompe, and would bypass the eventual therapeutic resistance often observed with ERT therapies. Both gene delivery and direct gene correction are viable therapeutic strategies in Pompe disease. Direct gene delivery is more practical given existing technologies because the field lacks a durable gene editing approach that could correct all Pompe disease mutations, a similar issue in treating DMD. Viral delivery of acid α‐glucosidase has been shown to clear glycogen and increase force production in patient specific induced pluripotent stem cell‐derived cardiomyocytes (Sato et al., 2015). Systemic delivery of an adenoviral construct with precursor human acid α‐glucosidase has also been shown to be a viable strategy for induction of acid α‐glucosidase production in the liver (Han et al., 2017) (NCT04093349) (Table 3). However, both ERT and AAV‐based gene delivery elicit an immune response where patient antibodies neutralise a significant portion of precursor acid α‐glucosidase (Bronsema et al., 2015). The key to advancing treatment in Pompe disease is therefore the durable delivery of a therapy in a single dose or averting re‐delivery resistance.

**TABLE 3:** Completed and ongoing clinical trials for Pompe disease.

Trial number	Intervention	Findings	Limitations	Ref.
NCT02782741	M6P residue‐enhanced ERT	Increased respiratory function, improved mobility endurance compared to treatment with non‐M6P residue‐enhanced ERT	No cardiac outcomes measured	Diaz‐Manera et al. (2021)
NCT01924845	ERT (recombinant acid α‐glucosidase)	Improved respiratory function, improved mobility endurance	Study terminated, additional data published without statistical analysis	Byrne et al. (2017), Hiwot et al. (2015)
—	Combined chaperone and ERT (*N*‐butyldeoxynojirimycin plus acid α‐glucosidase)	Increased acid α‐glucosidase activity with chaperone compared to without, unchanged functional measurements	Very small patient cohort	Parenti et al. (2014)
NCT02240407	Gene therapy with immunomodulation (AAV‐mediated transfer of *GAA* gene plus rituximab and sirolimus)	Completed, no results reported	—	—
NCT00976352	Gene therapy (AAV‐mediated transfer of *GAA* gene)	Improved respiratory outcomes (tidal volume)	Very small patient cohort	Smith et al. (2013)
NCT04093349	Gene therapy (liver targeted AAV‐mediated delivery of *GAA* gene)	Currently ongoing, no results reported	—	—

## 6 THE PRACTICALITIES AND HURDLES OF DELIVERING DURABLE GENE THERAPIES ACROSS GENETIC MUSCLE CONDITIONS

Individual musculopathies are rare, but as a group of conditions they affect ∼1 in 1500–3000 individuals. They are progressive multi‐tissue pathologies with diverse genetic drivers and their cardiac phenotypes are varied but frequently present with fibrosis, cell death and heart failure. Small molecule therapies have utility treating these conditions as they can be systemically delivered and are often bioavailable across a variety of disease‐affected tissues. This comes at the expense of promiscuity of small molecule binding, which can cause side effects limiting therapeutic window and adherence. They are also contraindicated in certain patient groups. This is where biological therapies that directly target genetic pathomechanisms could be used to provide expanded precision treatment options. The contemporary genetic understanding of musculopathies, and the availability of genetic tools, is allowing the development of novel targeted biological therapies. These biological therapies aim to prevent and reverse disease progression. We discuss the feasibility and hurdles that need to be overcome to allow the delivery of gene therapies across musculopathies that have diverse genetic drivers.

There are a number of methodological considerations to take into account when designing a gene therapy. For example, Pompe disease is caused by a lack of functional GAA protein in the cell, and the *GAA* gene is small enough to package into an AAV that can be efficiently targeted to the liver. Therefore, direct gene replacement is a tractable approach for Pompe disease. Using gene replacement also overcomes the need to specifically correct rare variants that drive haploinsufficiency of GAA. On‐going clinical trials are currently assessing the safety and durability of such an approach using AAV‐*GAA* delivery to the liver in early‐ and late‐stage Pompe disease. Initial non‐human primate (NHP) experiments have shown dose‐dependent expression of GAA in plasma with no abnormal histopathological findings after single doses of AAV*‐GAA* (Armour et al., 2019). This finding suggests that the AAV‐*GAA* construct is able to increase circulating plasma GAA levels. It will be useful to understand the durability of the GAA response and if this level of plasma GAA translates to therapeutic efficacy in affected tissues. Importantly, phase I/II trials are testing the safety of AAV‐*GAA* by monitoring immune responses to the AAV capsid and *GAA* transgene. This is being done to understand possible immunogenicity of the construct in the context of the sustained expression of a transgene. While studies in NHPs have shown high initial hepatocyte transduction, there is still <1% long‐term transduction observed, despite 10% of cells retaining vector DNA (Greig et al., 2022). However, initial findings in mice have shown that direct delivery of AAV‐*GAA* to specific tissues can catalyse the breakdown of glycogen, suggesting that there is reversibility of glycogen accumulation, which is encouraging. It is important to define the delivery window for a gene therapy especially in the case of Pompe disease, which drives progressive fibrosis and may not be reversed by re‐establishing GAA levels alone. This speaks to a broader set of open questions in the field relating to the optimal interventional window across inherited conditions that can either prevent the onset or allow sufficient reversal of pathology to benefit patient outcomes.

In the case of DMD, direct gene delivery is hampered by the size of dystrophin, which is too large to be packaged into existing viral vectors. However, delivery of micro‐dystrophins may be sufficient to partially restore cellular function and treat disease. Experiments have tested the efficacy of delivering a small dystrophin fragment called micro‐dystrophin 5 (μDys5). μDys5 has been packaged into an AAV (AAV‐μDys5) and injected into DMD mice at 4 weeks of age, which prevented fibrotic and inflammatory processes in the heart up to 18 months of age (Piepho et al., 2023). Micro‐dystrophins are unlikely to fully replace the function of full‐length dystrophin inside a DMD cell, so it may be necessary to increase the amount of full‐length or near full‐length dystrophin to treat DMD. This is where ASOs show promise, as they can be used to increase full‐length dystrophin expression by skipping disease‐causing DMD exons. This is the mechanism of action for Exondys 51, an FDA‐approved ASO for patients carrying the DMD exon 51 allele. However, exon 51 variants are only seen in ∼13% of DMD patients, so other gene therapies or additional exon‐specific ASOs will be needed to treat DMD variants outside of exon 51.

When designing and testing a gene replacement therapy it is important to understand the level of re‐expression that is necessary to prevent or slow disease progression across affected tissues. This is especially true for DMD where 4–20% of full‐length dystrophin re‐expression in the diaphragm can ameliorate DMD pathology, but other tissues and muscle groups may require up to 70% re‐expression. AAV delivery is an attractive approach for DMD as it can produce sustained expression across a variety of affected tissues. However, robust delivery to the heart is still a significant hurdle. Novel viral serotypes are being developed that can de‐target the liver where most AAV is cleared. This will decrease liver toxicity and decrease the viral titres that will need to be systemically delivered to target disease‐relevant tissues outside of the liver, making treatments safer. Another approach to increase AAV delivery to the heart is to use direct heart‐targeted molecular cardiac surgery with recirculating gene delivery (MCARD) (Katz et al., 2010). MCARD is an invasive procedure, but may be the most suitable cardiac delivery method currently available. Future developments in vector design aim to establish a therapy for DMD which would be a combination of systemically delivered liver de‐targeted AAV serotypes with specific heart or muscle targeting. This would allow dual delivery of AAV to efficiently target multiple disease‐affected tissues (Davis et al., 2023). Tissue specificity of delivery is also a key hurdle to developing ASOs for DMD where significant effort is being employed to develop novel ASO chemistries that bias tissue specific uptake (Roberts et al., 2020). This technological advance would allow the deployment of admixtures of ASOs to ensure effective delivery to multiple disease relevant tissues (Gagliardi & Ashizawa, 2021).

Fredrich's ataxia is a complicated multisystem condition which, much like DMD and Pompe disease, needs a treatment or treatments that can target multiple tissues. Theoretically Friedrich's ataxia could be treated by direct gene delivery as the *FXN* gene is small enough to be virally packaged. However, it has been shown that frataxin over‐delivery can be potentially toxic to the cells (Belbellaa et al., 2020). Further complicating the use of direct gene delivery as a therapy is the lack of AAV serotypes that can cross the blood–brain barrier and efficiently infect relevant CNS tissues (Meseck et al., 2022). This can be overcome by the use of higher viral titres, but higher multiplicities of infection may then instead drive frataxin overexpression toxicity. A potential avenue to overcome this issue is the design of low‐efficiency promoters. These promoter elements could limit gene expression from a single viral entry of the cell and thereby reduce the likelihood that a high cellular viral delivery would cause large increases in frataxin that cause pathology.

Another key approach being tested for treating Friedrich's ataxia is the delivery of genome engineering constructs employing CRISPR/Cas‐9. Here the editing strategy would aim to remove the expanded repeat elements in the *FXN* gene to restore endogenous allelic frataxin expression. This would circumvent the risk of overexpression toxicity as *FXN* expression would be controlled by endogenous gene regulatory mechanisms. So far the use of Cas9 as a therapy has been limited by promiscuous off‐target Cas9 DNA cleavage causing double stranded breaks (DSB), with a subsequent risk of mutagenesis (Rees & Liu, 2018). However, newer editing systems such as base editors and prime editors have been designed to circumvent DSBs and limit off‐target mutagenesis. Specifically, editors such as cytosine base editors (CBEs) or adenine base editors (ABE) use a catalytically inactive Cas9‐tethered deaminase that catalyses a single base change within the base editor target window. These CBEs and ABEs reduce the likelihood of DSBs but can still cause bystander edits, which are single base changes that are catalysed by the promiscuity of the base editing complex within the vicinity of the target nucleotide. Base editing strategies could be used to correct premature stop codons in DMD and Pompe disease. However, Friedrich's ataxia requires the removal of an expanded repeat element, which is where prime editors may provide a more tractable editing strategy. This is because prime editors mediate donor‐free precise DNA editing to produce all possible transition/transversion mutations and, importantly, allow discrete small insertions or deletions (Anzalone et al., 2019). Prime editing can also allow base edits up to 34 base pairs from the NGG protospacer adjacent motif extending its utility to 90% of genetic disorders (Scholefield & Harrison, 2021). These editors could have significant impact in many genetic disease settings in the future once there is proof of efficacious on‐target editing with minimal off‐target and bystander editing. Collectively, these editing approaches provide a promising set of tools for direct therapeutic targeting of the genome, which may only require a single ‘one and done’ delivery (Musunuru et al., 2021).

The therapeutic use of genome editors is still ultimately limited by the available delivery modalities that can package the editing modality and provide targeted tissue delivery. Dual AAV systems may be needed to deliver all the necessary components of prime editors, but their infectivity of the heart is still low (∼11%) (Davis et al., 2023). Alternatives such as lipid nanoparticles still have a predominant hepatic uptake, which limits heart/muscle delivery, but novel lipid nanoparticle targeting strategies could allow precise tissue targeting, which would aid delivery to tissues of interest in musculopathies. Clearly, delivery is still one of the major practical hurdles that need to be overcome to unlock the potential of human gene therapies. The field may have to think more creatively about solving the delivery bottleneck, as demonstrated in a study that has hijacked novel pathogen biology to provide a tuneable and robust delivery system for multiple human tissue types (Kreitz et al., 2023).

In the 20 years since the first approved genetic therapy, 33 gene therapies have been approved, and there are thousands of on‐going clinical trials exploring novel genetic approaches across a wide variety of diseases (Shahryari et al., 2021). However, cardiovascular disease is still relatively under‐represented in on‐going clinical trials of gene therapies. The lessons learned in other disease areas are providing a blueprint for advancing cardiovascular gene therapies. There is hope that these lessons will then de‐risk and accelerate the development of targeted gene therapies in rarer multi‐system conditions that exhibit cardiac pathologies.

## AUTHOR CONTRIBUTIONS

Yiangos Psaras and Christopher Toepfer were involved in the conception of the review, drafting of the work, revising, and critically appraising the content. Both authors have read and approved the final version of this manuscript and agree to be accountable for all aspects of the work in ensuring that questions related to the accuracy or integrity of any part of the work are appropriately investigated and resolved. All persons designated as authors qualify for authorship, and all those who qualify for authorship are listed.

## CONFLICT OF INTEREST

The authors declare they have no conflicts of interest.
